# Biological process engineering by *Pseudomonas* sp. ABC1: a discovery-stage ecological chassis for open bioproduction

**DOI:** 10.3389/fbioe.2026.1926795

**Published:** 2026-10-26

**Authors:** Daniel Valenzuela-Heredia, Sebastián Fuentes-Alburquenque, Carla Trigo, Carlos Henríquez-Castillo

**Affiliations:** 1 Escuela de Química y Farmacia. Centro de Investigación en Recursos Naturales y Sustentabilidad (CIRENYS). Universidad Bernardo O’Higgins, Santiago, Chile; 2 Escuela de Medicina, Facultad de Ciencias de la Salud, Universidad del Alba, La Serena, Chile

**Keywords:** extracellular polysaccharides, microbial consortium, next-generation industrial biotechnology, open cultivation, pellicle biofilm, *Pseudomonas* sp. ABC1, siderophores

## Abstract

**Introduction:**

Conventional industrial biotechnology remains constrained by contamination control requirements and energy-intensive biomass separation. Next-generation industrial biotechnology (NGIB) seeks microbial platforms that retain useful production phenotypes under open cultivation while enabling simpler process configurations. Here, we evaluated *Pseudomonas* sp. ABC1, a fluorescent siderophore-producing bacterium, as a discovery-stage ecological chassis for open bioproduction.

**Methods:**

Seven-day open, non-sterile static cultivation was integrated with community profiling, microscopy, elemental analysis, peptidomics, proteomics, and comparative genomics.

**Results:**

ABC1 formed a cohesive floating pellicle associated with a non-axenic bacterial consortium that included *Pseudomonas, Delftia, Flavobacterium, Acidovorax*, and additional environmental genera. Genome-informed reconstruction of representative community members indicated distributed capacities for iron acquisition, oxidative-stress protection, adhesion, nutrient acquisition, and extracellular-matrix metabolism. LC-MS/MS recovered 1,518 *de novo* peptide sequences, including 1,478 non-redundant sequences, with recurrent N-hydroxy-cyclo-ornithine derivatives and other modified peptide forms. These signatures are consistent with metallophore-associated biosynthesis predicted from the extensive iron-acquisition machinery previously described for ABC1. Comparative genomics further showed that ABC1 lacks complete canonical Pel, Psl, alginate, and PNAG systems but contains two extended loci encoding complementary functions for non-canonical extracellular-polysaccharide assembly, including rfbDCAB precursor synthesis, multiple glycosyltransferases, candidate Wzx/Wzy-like proteins, and export-associated components. ABC1 also encodes an expanded nutrient-acquisition architecture and 29 cyclic-di-GMP-associated proteins. Calcium-enriched pellicle microdomains and extracellular adhesive proteins provide additional candidate structural features, although mineral identity and polymer composition remain unresolved.

**Discussion:**

Collectively, these data support ABC1 as a promising discovery-stage system in which ecological selection, extracellular-matrix organization, and biosynthetic diversification can be investigated under open cultivation. Quantitative validation of productivity, contamination resistance, biomass-recovery performance, safety, and scale-up remains necessary before industrial advantage can be established.

## 1 Introduction

Industrial biotechnology provides renewable routes to chemicals, materials, and other bioproducts while reducing dependence on fossil feedstocks (Etit et al., 2024; Nielsen et al., 2022). Nevertheless, conventional industrial biotechnology remains constrained by contamination-control requirements, process complexity, and energy demand, particularly during upstream sterilization and downstream clarification (Liu et al., 2025; Janković et al., 2024). Next-generation industrial biotechnology (NGIB) addresses these constraints through robust microbial hosts, low-cost feedstocks, reduced freshwater demand, and process configurations compatible with open or minimally sterilized operation (Chen and Jiang, 2017; Yu et al., 2019). Established NGIB development has been led by extremophilic chassis such as Halomonas spp., whereas conventional industrial biotechnology also relies on extensively engineered bacterial and yeast hosts supported by quantitative strain- and process-performance data (Yu et al., 2019; Ye and Chen, 2021; Nielsen et al., 2022). ABC1 is therefore evaluated here as a biologically distinct candidate ecological chassis rather than as a quantitatively superior replacement for established platforms.

Upstream and downstream operations are tightly coupled in microbial manufacturing. Contamination control can impose substantial thermal and operational costs, whereas downstream recovery commonly requires cell removal, concentration, and purification steps that can dominate process economics (Guajardo and Schrebler, 2023; Janković et al., 2024). Microbial aggregation, flocculation, and biofilm formation can physically reorganize a culture and may reduce the solids burden entering clarification, but such benefits are process-specific and require direct engineering measurements (Yang et al., 2024). Open-culture systems therefore provide an opportunity to investigate how ecological competition, matrix formation, and spatial organization influence the persistence of a production phenotype.

*Pseudomonas sp*. ABC1 (NCBI RefSeq assembly GCF_013395055.1) is a mucoid fluorescent pseudomonad previously characterized for an unusual investment in iron homeostasis, transport, acquisition, and regulation, representing approximately 5% of its genome (Valenzuela-Heredia et al., 2021). Its genome also encodes multiple non-ribosomal peptide synthetase systems associated with siderophore biosynthesis, including an Azotobacter-related pyoverdine-like pathway and a yersiniabactin-type iron-chelation system. These features make ABC1 an informative model for examining whether strong nutrient-acquisition capacity can coexist with stable extracellular organization in an open microbial culture.

Under static cultivation, ABC1 forms a robust pellicle at the air-liquid interface. Pellicles are structured microbial communities in which motility, attachment, extracellular polymers, nutrient gradients, and cyclic-di-GMP signaling can jointly control the transition from planktonic to sessile growth (Madsen et al., 2015; Martin et al., 2016). In *Pseudomonas*, this architecture is commonly associated with Pel, Psl, alginate, or other extracellular-matrix components. The unusual mucoid phenotype of ABC1 despite the absence of complete canonical matrix systems prompted us to investigate whether alternative glycan-biosynthesis and regulatory architectures could explain its extracellular organization.

In this study, we integrated endpoint open-culture observations with microscopy, elemental analysis, 16S rRNA amplicon profiling, LC-MS/MS peptidomics/proteomics, and comparative genomics. We reconstructed candidate extracellular-polysaccharide loci, classified TonB-dependent receptor functions, quantified cyclic-di-GMP-associated domains, and estimated the genome-inferred functional potential of representative consortium members. The specific advance is biological and mechanistic: the study links pellicle formation and persistence in a non-axenic open community with candidate nutrient-acquisition, cyclic-di-GMP, and non-canonical glycan-assembly architectures. Our objective was not to validate a fermentation process or demonstrate industrial utility, but to define testable features of ABC1 as a discovery-stage ecological chassis for subsequent strain and process engineering.

## 2 Materials and methods

### 2.1 Bacterial strain and culture conditions

*Pseudomonas* sp. ABC1 was cultivated in acetate, ammonia, and mineral (AAM) medium as described previously (Valenzuela-Heredia et al., 2021). Cultures were refreshed with a 10% (v/v) inoculum and maintained as 7-day batch cultures under static conditions, without mechanical agitation or forced aeration, with the liquid surface exposed to the ambient laboratory atmosphere. Cultures were established in 5-L plastic bioreactors and incubated under ambient laboratory temperature conditions, approximately 10 °C at night and 20 °C during the day. All cultivations were performed in triplicate. Pellicle formation and the characteristic greenish-yellow coloration of the culture were recorded during the cultivation period. The source experiment was designed as an endpoint integrative characterization rather than as a controlled bioprocess-performance study; consequently, periodic sampling and longitudinal pH, dissolved-oxygen, biomass, substrate-conversion, titre, yield, and productivity profiles were not available. The open-culture experiment is therefore interpreted as a discovery-stage batch model rather than as a validated scale-up fermentation or a basis for quantitative industrial-performance claims.

### 2.2 Siderophore production and elemental analysis

The pellicle was harvested from the 7-day culture surface for subsequent analyses. Cell-free supernatant from the open static culture was obtained by centrifugation at 10,000 × g for 10 min and evaluated spectrophotometrically at 410 nm as an optical indicator associated with the fluorescent siderophore phenotype. Because a calibrated metabolite-concentration standard was not used, absorbance measurements are not reported as absolute siderophore concentration or productivity.

Microstructural and elemental characterization of the mature 7-day floating pellicle was performed by scanning electron microscopy (SEM). Pellicle sections were mounted on glass slides supported on aluminum stubs using carbon tape and gold-coated. SEM analyses were performed with a Zeiss EVO MA-10 microscope equipped with a tungsten filament gun. Energy-dispersive X-ray spectroscopy (EDX) was acquired using an Oxford Instruments system with a PentaFET detector at 20 kV and an 8-mm working distance. EDX was interpreted as elemental evidence.

### 2.3 Microbial consortium taxonomic characterization via partial 16S rRNA gene sequencing

The V4-V5 region of the 16S rRNA gene was used to characterize bacterial community composition within the pellicle formed after 7 days of static open culture. Total genomic DNA was prepared using the PowerSoil DNA Isolation Kit (QIAGEN) following the manufacturer’s protocol. Mechanical disruption was performed with a BeadBug six microtube homogenizer (Benchmark Scientific) using four 90-s cycles at 4,350 rpm. Purified DNA was used as template for PCR amplification with primers 515F-Y (GTGYCAGCMGCCGCGGTAA) (Parada et al., 2016) and 926R (CCGYCAATTYMTTTRAGTTT) (Quince et al., 2011).

This bacterial/archaeal primer pair targets the V4-V5 region and incorporated Illumina adapters. Amplicons were multiplexed and sequenced on the Illumina MiSeq platform (2 × 250 bp) at Argonne National Laboratory (Lemont, IL, USA), following the sequencing framework described by Caporaso et al. (2012). Demultiplexed reads were quality filtered, merged, and chimera filtered with DADA2 to infer amplicon sequence variants (ASVs) (Callahan et al., 2016). Taxonomy was assigned against SILVA release 138 (Quast et al., 2012) using VSEARCH (Rognes et al., 2016). Community analyses were performed in QIIME 2 (Bolyen et al., 2019).

### 2.4 Proteomic and *de novo* peptidomic profiling

*Pseudomonas* sp. ABC1 was maintained for 7 days in open culture. Floating pellicle biomass was physically separated from the medium using sterile tweezers. The remaining supernatant, containing soluble extracellular proteins and naturally occurring peptides, was loaded onto Amberlite XAD resin (Sigma-Aldrich) and eluted with analytical-grade methanol. Proteins in the methanolic fraction were treated with denaturing detergents and chaotropic agents and subjected to tryptic digestion. The resulting peptides were desalted using Sep-Pak C18 cartridges (Merck-Millipore), dried under vacuum, and quantified by infrared spectroscopy. Approximately 500 ng of tryptic digest were injected into a nanoElute nanoHPLC coupled to a timsTOF Pro mass spectrometer (Bruker Daltonics) using a 25 cm × 75 μm C18, 1.6 μm Aurora column (IonOpticks). Peptides were separated with a 2%–38% acetonitrile gradient over 90 min and acquired using data-dependent acquisition (DDA).

Raw MS/MS spectra were processed in PEAKS Studio X (Bioinformatics Solutions Inc.) against a compiled *Pseudomonas* spp. UniProt database together with *de novo* sequence reconstruction. Because the analyzed extracellular fraction originated from a mixed open culture and peptide-level taxonomic resolution was not sufficient to uniquely assign all conserved peptides, the dataset is described throughout as the open-culture secretome/peptidome rather than as an ABC1-exclusive secretome. Peptide modifications are interpreted as chemical signatures consistent with candidate biosynthetic processes and not as structural identification of specific metabolites.

### 2.5 Comparative bioinformatics

The complete ABC1 genome was compared with *Pseudomonas aeruginosa* PAO1, *Pseudomonas protegens* Pf-5 and CHA0, *Pseudomonas fluorescens* SBW25, *Pseudomonas putida* KT2440, *Pseudomonas brassicacearum* NFM421, *Pseudomonas chlororaphis* 30–84, *Azotobacter vinelandii* DJ, and *Acetobacter tropicalis* NBRC 101654. Candidate extracellular-polysaccharide loci were reconstructed from annotated glycosyltransferase, precursor-synthesis, membrane, export, modification, and regulatory genes; genomic neighborhoods were extended around core hits and ranked by functional composition. Protein homology, amino-acid identity, query coverage, genomic colocalization, predicted transmembrane helices, and Pfam domains were integrated. Canonical Pel, Psl, alginate, cellulose, and PNAG modules were screened separately. The *A. tropicalis* comparison was retained as a biochemically characterized polysaccharide-system reference rather than as the nearest evolutionary relative; phylogenetic and functional conclusions were therefore based primarily on the *Pseudomonas* comparator set.

TonB-dependent beta-barrel proteins were identified by Pfam/HMM screening and curated against annotated receptor families. Cyclic-di-GMP architecture was summarized from GGDEF, EAL, and HD-GYP domains. The exploratory consortium reconstruction used representative proteomes for *Pseudomonas, Delftia, Acidovorax, Adhaeribacter, Pedobacter, Flavisolibacter,* and *Rufibacter*. Proteins were assigned to 16 predefined functional modules, normalized per 1,000 predicted proteins, and weighted by genus-level relative abundance derived from the amplicon profile. A complementarity index was calculated as one minus the fraction of abundance-weighted functional potential contributed by the dominant representative taxon. Because 16S-defined genera may contain substantial strain-level variation, this analysis estimates genome-inferred potential and does not demonstrate *in situ* transcription, flux, metabolite exchange, or causal ecological interactions.

## 3 Results

### 3.1 Open cultivation and calcium-enriched pellicle microdomains

Seven-day static open cultivation produced a cohesive floating pellicle at the air-liquid interface (Supplementary Figure S1). SEM-EDX revealed spherical microdomains embedded within the pellicle that were enriched in elemental calcium relative to smoother matrix regions (Figure 1; Supplementary Figure S2). Calcium represented approximately 4% of the bulk pellicle signal and increased to >10% within selected spherical microdomains, whereas phosphate decreased to <1%. These observations demonstrate localized calcium enrichment within the matrix. The elemental pattern is compatible with, but does not establish, a carbonate-rich phase; mineral identity requires confirmation by phase-specific methods such as X-ray diffraction or Raman spectroscopy. Accordingly, these structures are referred to here as calcium-enriched microdomains rather than as confirmed microbially induced calcium carbonate precipitates.

**FIGURE 1:**
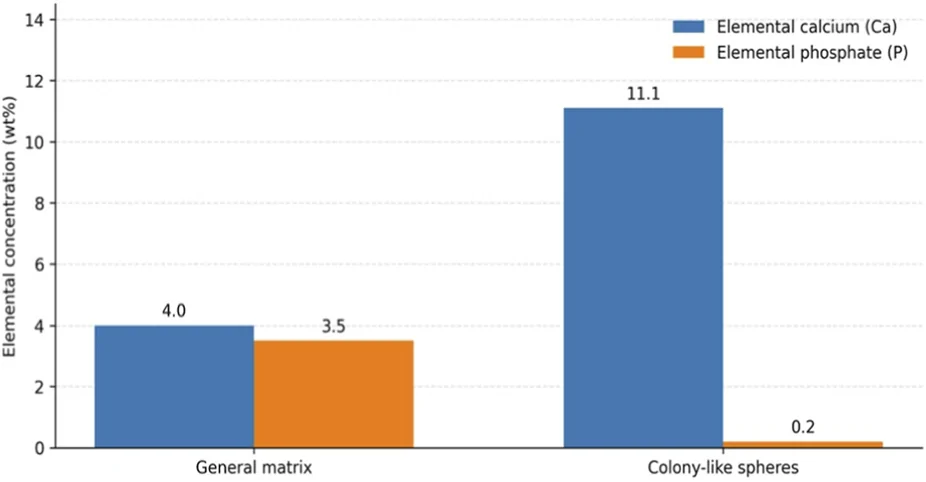
EDX-based elemental comparison between the bulk pellicle matrix and selected calcium-enriched microdomains. Calcium increased from approximately 4 wt% in the general pellicle matrix to >10 wt% in selected microdomains, whereas phosphate decreased to <1 wt%. Values correspond to the endpoint elemental measurements shown. EDX provides elemental composition and does not establish mineral phase or biological causality; corresponding SEM images and elemental maps are provided in Supplementary Figure S2.

### 3.2 Community structure and genome-informed functional potential

Amplicon profiling of the mature 7-day pellicle showed that open cultivation produced a non-axenic assemblage containing *Pseudomonas, Delftia, Flavobacterium, Acidovora*x, and additional environmental genera (Supplementary Table S1; Supplementary Figure S3). The data therefore support the persistence of ABC1 within a mixed community rather than the formation of an exclusive or axenic population. Because the available dataset represents an endpoint community profile, temporal succession and causal selection cannot be resolved from these data alone.

Genome-informed reconstruction across seven representative taxa indicated broadly distributed capacities for iron acquisition, TonB-dependent uptake, EPS/glycan metabolism, cyclic-di-GMP signaling, motility, oxidative-stress protection, and nutrient acquisition, with distinct abundance-weighted contributions (Figure 2). *Pseudomonas* contributed strongly to predicted iron-acquisition potential, whereas *Delftia* showed comparatively high contributions to motility and chemotaxis, cyclic-di-GMP signaling, adhesion, secretion, phosphorus acquisition, and oxidative-stress protection. Bacteroidota representatives, including *Adhaeribacter, Pedobacter, Flavisolibacter*, and *Rufibacter,* contributed substantially to TonB-dependent uptake and glycan/polymer-associated functions. The highest complementarity indices were observed for phosphorus acquisition (0.751), cation homeostasis/biomineralization-associated functions (0.736), iron acquisition (0.714), oxidative-stress protection (0.713), TonB-dependent uptake (0.712), and secondary-metabolite biosynthesis (0.684) (Figure 2). These values describe encoded functional distribution among representative genomes and should not be interpreted as direct evidence of metabolic exchange within the pellicle.

**FIGURE 2:**
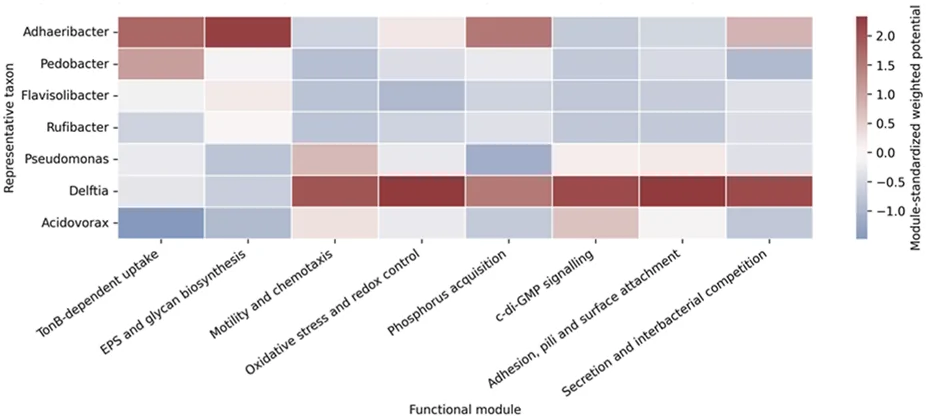
Exploratory abundance-weighted functional reconstruction of representative consortium taxa. Values were calculated from reference-proteome functional-module counts normalized per 1,000 predicted proteins and weighted by genus-level relative abundance. The analysis estimates genome-inferred functional potential and does not demonstrate *in situ* expression or metabolic exchange.

Axenic and mixed-culture extracellular fractions showed closely comparable absorbance/emission spectral profiles (Supplementary Figure S4). This observation indicates only that the characteristic fluorescent extracellular phenotype remained detectable in the mixed culture. Because the cultures were not evaluated in a matched quantitative process experiment, these endpoint optical profiles do not demonstrate equivalent or superior growth, metabolite production, titre, yield, productivity, contamination resistance, or process stability under open cultivation relative to sterile cultivation.

### 3.3 Open-culture secretome and peptidome

A total of 1,518 *de novo* peptide sequences were recovered from the liquid secretome dataset, yielding 1,478 non-redundant peptide sequences after redundancy filtering (Supplementary Table S1). The average peptide length was approximately 13 amino acids, with most sequences ranging tightly between 7 and 20 residues.

PTM analysis identified N-terminal acetylation, carbamylation, deamidation, citrullination, methionine oxidation, homoserine formation, and N-hydroxy-cyclo-ornithine derivatives (Table 1). Ornithine hydroxylation is a characteristic biosynthetic step in several hydroxamate-containing siderophores of fluorescent pseudomonads (Visca et al., 2007; Ge and Seah, 2006; Meneely and Lamb, 2007). The recurrence of N-hydroxy-cyclo-ornithine-containing peptide forms is therefore consistent with the iron-acquisition and pyoverdine-related biosynthetic potential encoded by ABC1. However, these peptide signatures do not constitute direct structural identification of a specific siderophore or establish that every detected peptide originated from ABC1.

**TABLE 1:** Post-translational modifications identified in the reconstructed peptidome of *Pseudomonas* sp. ABC1.

Post-translational modification	Number of peptides	Percentage (%)
Acetylation (Protein N-term)	293	19,3
Carbamylation	281	18,51
Deamidation (NQ)	80	5,27
Citrullination	72	4,74
Oxidation (M)	63	4,15
Homoserine	34	2,24
N-Hydroxy-cyclo-ornithine	32	2,11
Unmodified peptides	663	43,68
**Total**	**1518**	**100**

Proteomic characterization of the extracellular fraction additionally recovered motifs matching bacterial collagen-like proteins and filamentous-hemagglutinin-related proteins. These proteins are plausible contributors to adhesion and matrix organization, and their structural role in the ABC1 pellicle remains to be tested experimentally.

### 3.4 Comparative genomic reconstruction of pellicle-associated functions

In *Pseudomonas*, alginate, Psl, Pel, and other extracellular polymers contribute to biofilm architecture (Hentzer et al., 2001; Reichhardt et al., 2020; Gheorghita et al., 2023). We therefore first screened ABC1 for complete canonical matrix-biosynthesis systems before reconstructing alternative glycan-associated regions.

Canonical-locus screening confirmed the absence of complete pel, psl, alginate, and PNAG systems in ABC1. Instead, two high-ranking extended glycan regions were reconstructed. PSL_140 spans approximately 53.9 kb and contains 46 genes, including ten curated glycosyltransferases, four predicted polysaccharide-modification enzymes, two regulatory proteins, membrane-associated proteins, and an export-associated component. PSL_143 spans approximately 35.9 kb and contains 30 genes, including the rfbDCAB precursor module, two additional glycosyltransferases, membrane proteins, and candidates for Wzx/Wzy-dependent assembly (Figure 4; Supplementary Table S2). These extended regions may contain several transcriptional units; operon boundaries and direct involvement in pellicle formation cannot be inferred from gene proximity alone.

**FIGURE 3:**
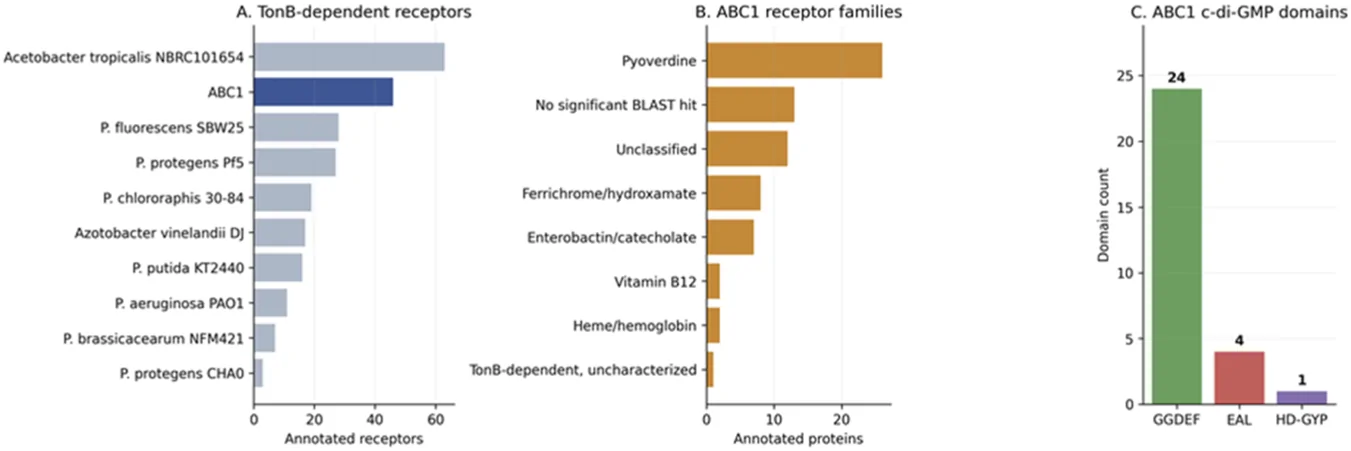
Expanded nutrient-acquisition and regulatory architecture of *Pseudomonas* sp. ABC1. **(A)** Stringently annotated TonB-dependent receptors across the comparative genome set. **(B)** Functionally classified ABC1 receptor families. **(C)** GGDEF, EAL and HD-GYP domain counts in ABC1. Domain-level HMM screening recovered additional TonB β-barrel candidates; counts shown in panels A–B are based on the curated annotation pipeline.

**FIGURE 4:**
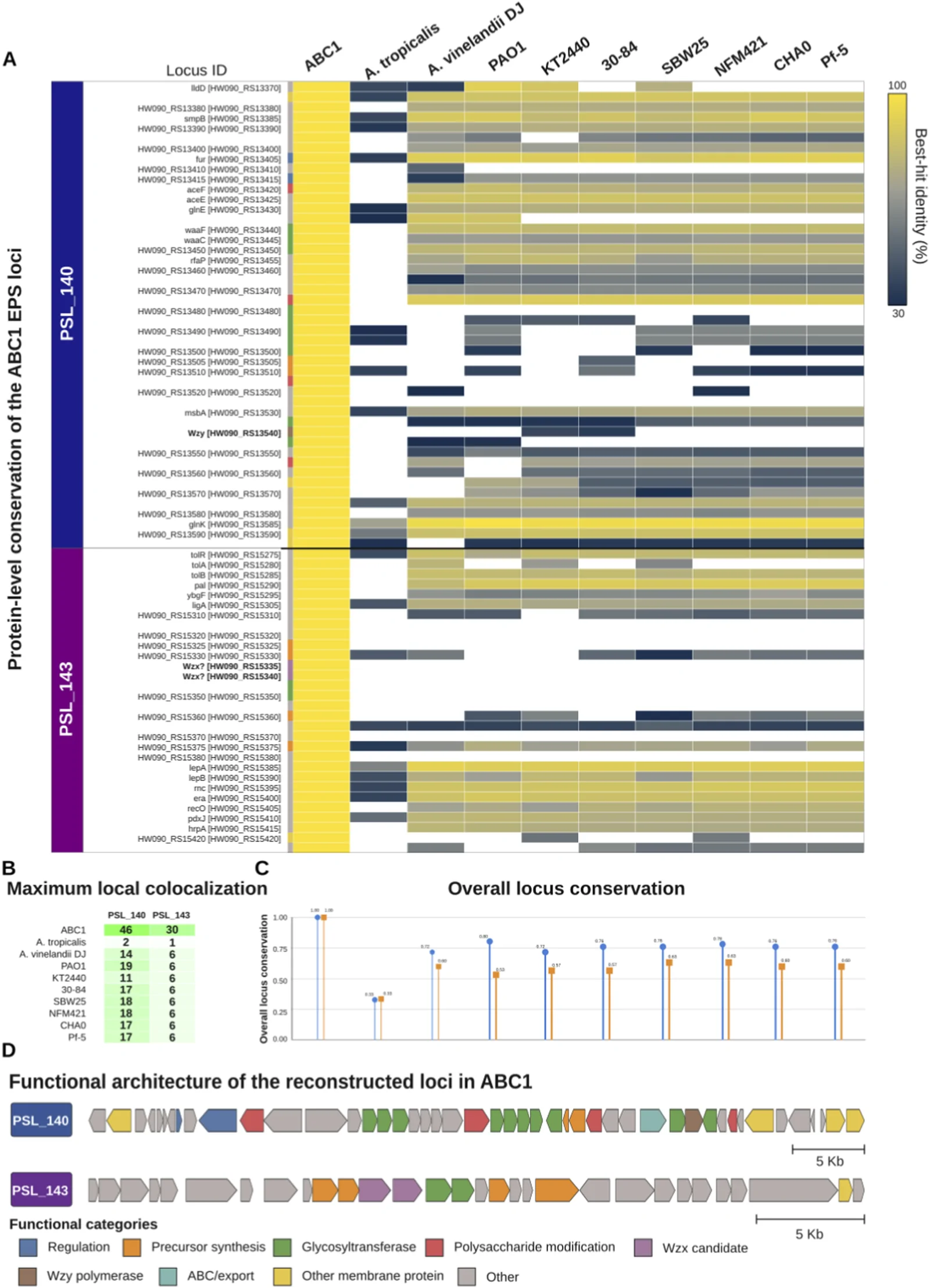
Comparative conservation, genomic colocalization, and functional architecture of the reconstructed extracellular-polysaccharide-associated loci in *Pseudomonas sp*. ABC1. **(A)** Protein-level conservation of representative genes from the two reconstructed ABC1 polysaccharide-associated regions, PSL_140 and PSL_143, across the comparative genome set. Rows correspond to ABC1 proteins and columns to comparator genomes. Cell color indicates the amino-acid identity (%) of the best-scoring homolog recovered for each ABC1 protein; ABC1 is shown as the reference. White cells indicate absence of a homolog meeting the analysis criteria. Genes are classified according to curated functional categories, including regulation, dTDP-sugar precursor biosynthesis, glycosyltransferases, polysaccharide modification, membrane translocation, polymerization, and export. Symbols highlight the rfb/rml core genes, candidate Wzx-like proteins, the Wzy-like polymerase, and selected regulatory proteins. PSL_140 and PSL_143 are internal identifiers assigned to the reconstructed ABC1 loci and do not denote the canonical *Pseudomonas* psl operon. **(B)** Maximum local colocalization of ABC1 homologs. Values indicate the largest number of homologs from each ABC1 locus recovered within a single local genomic neighborhood in the corresponding genome, thereby distinguishing conservation of individual proteins from conservation of locus-level organization. **(C)** Overall locus conservation, expressed as the fraction of ABC1 proteins within PSL_140 or PSL_143 for which homologs were detected in each comparator genome. **(D)** Genomic organization and curated functional composition of the complete PSL_140 and PSL_143 regions in ABC1. Arrows indicate coding sequence orientation and are scaled according to genomic position; colors correspond to the functional categories used throughout the figure. PSL_140 comprises 46 genes spanning approximately 53.9 kb, whereas PSL_143 comprises 30 genes spanning approximately 35.9 kb. The reconstructed architecture includes the contiguous rfbDCAB module involved in dTDP-L-rhamnose precursor biosynthesis, multiple glycosyltransferases, polysaccharide-modification enzymes, membrane-associated proteins, export functions, and candidate components of a Wzx/Wzy-dependent assembly pathway. HW090_RS15335 was classified as the principal Wzx-like candidate based on its multi-pass transmembrane topology, polysaccharide-synthesis-associated Pfam domains, and locus context, whereas adjacent HW090_RS15340 was annotated as an O-antigen polymerase consistent with a Wzy-family polymerase. The combined conservation and colocalization patterns indicate that individual glycan-associated functions are broadly distributed among pseudomonads, while the complete ABC1 locus architecture is comparatively poorly conserved, consistent with genomic remodeling of polysaccharide-associated modules. Abbreviations: EPS, extracellular polysaccharide; GT, glycosyltransferase; rfb/rml, dTDP-L-rhamnose biosynthesis pathway; Wzx, putative polysaccharide repeat-unit flippase/translocase; Wzy, polysaccharide repeat-unit polymerase; ABC, ATP-binding cassette; DGC, diguanylate cyclase; GGDEF, diguanylate cyclase-associated domain; TM, transmembrane; kb, kilobase pairs.

Within PSL_143, HW090_RS15335 encodes a lipopolysaccharide-biosynthesis protein with ten predicted transmembrane helices and Polysacc_synt/Polysacc_synt_3 domains, producing the highest Wzx-like candidate score in the reconstruction. Adjacent HW090_RS15340 encodes a nine-transmembrane O-antigen polymerase consistent with a Wzy-family polymerase. Together with rfbDCAB and multiple glycosyltransferases, these proteins support a testable model for repeat-unit synthesis, membrane translocation, polymerization, and export; they are designated candidate components pending genetic and biochemical validation.

Protein-level comparison showed that individual ABC1-locus proteins are widely represented across pseudomonads, whereas complete local colocalization is limited (Figure 4, panels B–C). For PSL_140, 71.7%–80.4% of proteins had homologues in most *Pseudomonas* comparators, while maximum local colocalization ranged from 11 to 19 of the 46 genes. For PSL_143, 53.3%–63.3% of proteins had homologues, but only six were maximally colocalized in each comparator. *Acetobacter tropicalis* showed approximately 33% protein-level coverage and minimal local colocalization for either extended ABC1 locus. Thus, the comparison supports remodeling of broadly conserved glycan-associated functions.

ABC1 also showed an expanded nutrient-acquisition and regulatory architecture. Stringent annotation identified 46 TonB-dependent receptors, including 26 pyoverdine-receptor-like proteins, consistent with the previously described investment in iron acquisition. Domain-level HMM screening identified 29 cyclic-di-GMP-associated proteins: 24 GGDEF-domain proteins, four EAL-domain proteins, and one HD-GYP-domain protein (Figure 3C). This repertoire provides genomic capacity for extensive regulation of attachment and sessile growth, although expression and causal contribution to pellicle formation were not measured in this study.

## 4 Discussion

The integrated evidence positions ABC1 as a discovery-stage ecological chassis for open bioproduction, not as a validated industrial strain. The value of the present study lies in linking four observations that can now be tested independently: formation of a cohesive air-liquid-interface pellicle, persistence within a mixed open community, extensive nutrient-acquisition and cyclic-di-GMP architectures, and reconstruction of non-canonical glycan-associated loci. Relative to established NGIB platforms, particularly Halomonas-based systems already supported by extensive strain-engineering and process-performance data (Yu et al., 2019; Ye and Chen, 2021), the present novelty of ABC1 lies in its ecological self-organization rather than in demonstrated process superiority. These features provide a mechanistic framework for future engineering and direct benchmarking against established hosts.

Calcium-enriched microdomains may contribute to matrix organization because divalent cations can cross-link extracellular polymers and alter biofilm cohesion (Flemming and Wingender, 2010; Kim et al., 2025; Yang et al., 2026). However, EDX resolves elemental composition rather than mineral phase. The observed calcium enrichment and low phosphate signal are compatible with several calcium-rich materials, including carbonate-associated structures, and therefore cannot be used to claim microbially induced calcium carbonate precipitation (De Muynck et al., 2010; Dhami et al., 2013). Phase-specific spectroscopy and controlled precipitation assays are required before biomineralization can be assigned a functional role in pellicle mechanics.

The peptidomic dataset indicates substantial chemical diversity in the extracellular fraction. N-hydroxy-cyclo-ornithine is particularly relevant because ornithine hydroxylation occurs in hydroxamate-containing siderophore pathways and is compatible with the Azotobacter-related pyoverdine machinery previously identified in ABC1 (Ge and Seah, 2006; Meneely and Lamb, 2007; Visca et al., 2007; Valenzuela-Heredia et al., 2021). Nevertheless, *de novo* peptide signatures and PTMs cannot substitute for targeted metabolomics, structural elucidation, or authentic standards. We therefore interpret these observations as signatures compatible with metallophore-associated biosynthesis rather than as structural identification of a specific siderophore or metallophore. Siderophores/metallophores and extracellular polysaccharides are established classes of biotechnologically relevant molecules; however, their industrial value in ABC1 cannot be assessed without compound-specific identification and quantitative production metrics.

Iron acquisition and oxidative-stress protection provide a coherent physiological context for the observed extracellular signatures. ABC1 encodes manganese- and iron-dependent superoxide dismutases, KatG catalase, cytochrome c551 peroxidases, and an unusually large TonB-dependent receptor repertoire (Valenzuela-Heredia et al., 2021). These systems could support growth in iron-limited environments while limiting oxidative damage associated with intracellular iron.

Extracellular adhesive proteins and cyclic-di-GMP signaling provide additional candidate mechanisms for pellicle cohesion. Filamentous hemagglutinins promote aggregation and surface attachment in related pseudomonads (Sun et al., 2016), and collagen-like proteins can contribute to extracellular architecture in diverse bacteria (Qiu et al., 2021). In ABC1, their detection in the extracellular fraction is consistent with a structural contribution, while the 29 cyclic-di-GMP-associated proteins provide substantial regulatory capacity for switching between motile and sessile states. Direct expression measurements and perturbation experiments will be required to establish which of these factors control the phenotype.

Open cultivation produced a mixed consortium rather than an axenic ABC1 population. Genome-informed reconstruction suggests that representative members possess complementary encoded capacities for iron uptake, oxidative-stress protection, phosphorus acquisition, cation homeostasis, adhesion, and extracellular-matrix metabolism. This pattern is compatible with ecological filtering and division of labor but does not prove either process. In particular, *Delftia* is of interest because members of this lineage produce metallophores such as delftichelin A (de Kruijff et al., 2026), while Bacteroidota representatives encode numerous glycan-associated functions. The present analysis therefore generates explicit hypotheses about community function that can be tested with isolate-resolved genomics, metagenomics, transcriptomics, and pairwise competition experiments. However, iron competition assays, receptor expression, and siderophore-activity measurements are required to determine whether iron acquisition actually structures the open consortium.

The genomic reconstruction provides a stronger basis for interpreting the pellicle phenotype. ABC1 lacks complete canonical Pel, Psl, alginate, and PNAG systems but contains two extended regions that collectively encode precursor synthesis, multiple glycosyltransferases, membrane-translocation candidates, polymerization, modification, and export-associated functions. Their fragmented conservation across comparator pseudomonads argues for evolutionary remodeling of broadly conserved glycan modules rather than a simple one-to-one replacement by an *Acetobacter* pol locus. Candidate Wzx/Wzy-like proteins and the rfbDCAB module now provide defined targets for gene disruption, expression analysis, polymer purification, and compositional characterization. These loci therefore define testable candidate mechanisms for the ABC1 pellicle phenotype, but the present genomic reconstruction does not establish a causal role in pellicle formation.

Published microbial systems provide useful qualitative context but not a direct quantitative benchmark for ABC1 (Table 2). Controlled siderophore studies quantify pyoverdine or siderophore units, while extracellular-polymer and related bioproduct studies use distinct analytical definitions, hosts, media, and reactor configurations. Open non-sterile systems additionally rely on selective physicochemical conditions not evaluated here. Because ABC1 lacks compatible titre, yield, productivity, and kinetic data, cross-system values are presented only to define the measurements and controls required for subsequent process validation.

**TABLE 2:** Qualitative benchmarking of *Pseudomonas* sp. ABC1 against representative siderophore-producing, *Pseudomonas* bioproduction, and open non-sterile microbial systems.

Organism/strain	Product	Relevance to ABC1	Cultivation mode/scale	Reported titre/productivity	Key process conditions	Quantification basis/References
*Pseudomonas* sp. ABC1	Fluorescent siderophore phenotype; pyoverdine-like/metallophore-associated extracellular fraction (structure unresolved)	Study system	Open, non-sterile static batch; 5-L laboratory bioreactor	Titre: NR; productivity: NR. Optical fingerprint: λex,max ≈397 nm; λem,max ≈466 nm	Non-sterile tap water supplemented with acetate, ammonia, and mineral (AAM) components; 10% (v/v) inoculum; 7 d; static; no mechanical agitation or forced aeration; liquid surface exposed to ambient laboratory atmosphere; uncontrolled temperature following ambient day–night fluctuations (∼10 °C–20 °C)	Steady-state fluorescence spectroscopy, genomic and peptidomic evidence supports pyoverdine/metallophore-associated biosynthesis but does not establish structural identity or absolute concentration. This study
*Pseudomonas* sp. ANT_H12B	Pyoverdine	Direct pyoverdine comparator	Axenic/sterile cultivation. Aerobic shaken batch; 96-well optimization with 200-mL verification	223.50–512.60 µM pyoverdine; maximum 512.60 µM in GSA medium. Biomass-normalized production in GSA: 945.76 µM g−1 biomass	3 d; pH 7.0; 10 °C; 150 rpm; initial OD600 = 0.06; multiple carbon/nitrogen media and C:N ratios evaluated	CAS assay for screening; HPLC quantification with commercial *P. fluorescens* pyoverdine as standard. Musialowski et al. (2023)
*Pseudomonas aeruginosa* RZS9	Siderophore(s)	*Pseudomonas* siderophore scale-up comparator	Axenic/sterile cultivation. Shake-flask optimization followed by 5-L laboratory bioreactor	69.48% siderophore units (SU) after 24 h in the 5-L bioreactor; not a mass titre. Productivity: NR	Succinate medium; succinic acid 4.9 g L−1; pH 7.08; 27.8 °C; 24 h	Chrome Azurol S (CAS) shuttle assay at 630 nm; result expressed as % siderophore units. Shaikh et al. (2016)
*Azotobacter vinelandii* DJ	Azotobactin (pyoverdine-type siderophore)	Chemically related siderophore comparator	Axenic/sterile cultivation. Aerobic shaken batch culture	Approximately 25–50 mg L−1 azotobactin; productivity: NR	Modified Burk’s siderophore-production medium (SPB), no added Fe; 58 mM sucrose; 30 °C; 200 rpm; 72 h	Spectrophotometric estimate from A380 at pH 4.0 using ε = 23,500 M−1 cm−1 and approximate MW ≈ 1370 g mol−1. Villa et al. (2014)
*Pseudomonas mendocina*	Alginate (extracellular polysaccharide)	Extracellular polymer benchmark	Axenic/sterile cultivation. pH-controlled batch and continuous culture	≈20 g L−1 alginate in batch; similar concentration under N-limited continuous culture. >60% of utilized glucose converted to alginate under N limitation	Batch: 50 g L−1 glucose. Continuous: N limitation, D = 0.05 h−1; optimum dissolved-O2 tension ≈40 mmHg; alginate not produced under O2 limitation	Extracellular polysaccharide/alginate concentration measured in fermentation cultures. Sengha et al. (1989)
*Pseudomonas aeruginosa* O-2-2	Rhamnolipids (secreted glycolipid biosurfactants)	Secreted-product process-intensification benchmark	Axenic/sterile cultivation. 5-L batch followed by pH-stage-controlled fed-batch fermentation	24.06 g L−1 in batch; 70.56 g L−1 in pH-stage-controlled fed-batch. Volumetric productivity not reported in a directly comparable form	Soybean-oil-based fermentation medium; 30 °C; 500 rpm; pH-stage control; fed-batch process	Rhamnolipid titre measured after fermentation/product recovery. Zhu et al. (2012)
*Pseudomonas putida* LS46	Medium-chain-length PHA (intracellular biopolymer)	High-productivity *Pseudomonas* process benchmark	Axenic/sterile cultivation. High-cell-density, O2-limited fed-batch; 7-L bioreactor	17.7 ± 4.8 g L−1 mcl-PHA; final volumetric productivity 0.66 ± 0.14 g L−1 h−1	Octanoic acid carbon source; reactive pulse feeding using CO2/DO feedback; O2 limitation from ∼14 h; 27-h cultivation; atmospheric aeration	Intracellular PHA quantified relative to biomass and as volumetric polymer concentration. Blunt et al. (2019)
*Bacillus* sp. 2–6	L-lactic acid	Open, non-sterile process benchmark	Open fermentation without sterilization; 5-L batch and up to 30-L fed-batch	118.0 g L−1 and 4.37 g L−1 h−1 in 5-L batch; 182.0 g L−1 and 3.03 g L−1 h−1 in 30-L fed-batch	Thermophilic cultivation (∼50 °C); 10% (v/v) inoculum; 200 rpm; pH 5.6; no aeration; fermentation conducted openly without sterilization	L-lactic acid quantified using a biosensor analyzer and chiral HPLC for optical purity. Qin et al. (2009)

Table NR, not reported or not available as a compatible quantitative process metric; SU, siderophore units. Values are retained in the units and analytical framework of the original studies and are intended for qualitative benchmarking, not direct quantitative ranking. *Bacillus* sp. 2–6 is included specifically as a process comparator because production was explicitly conducted under open, non-sterile conditions; its product is not chemically comparable to the ABC1 siderophore-associated phenotype.

From a process perspective, the pellicle creates an observable spatial separation between aggregated biomass at the air-liquid interface and the underlying liquid fraction. This configuration motivates, but does not validate, the hypothesis that pellicle formation could reduce solids loading during clarification or enable alternative biomass-removal strategies. Similarly, the endpoint optical profiles indicate only that the characteristic ABC1-associated extracellular phenotype remained detectable in the mixed culture. Because no matched sterile-versus-open measurements of growth, substrate conversion, product titre, yield, productivity, contamination dynamics, or process stability were performed, the present study does not demonstrate that open cultivation performs equivalently to or better than sterile cultivation. Any proposed process benefit should therefore be considered a testable hypothesis requiring controlled engineering validation.

### 4.1 Limitations and future validation

A major experimental limitation of the present study is its endpoint design and the absence of matched sterile-versus-open process-performance measurements. Production kinetics, time-resolved biomass accumulation, substrate conversion, product titre, yield, volumetric productivity, contamination dynamics, and process stability were not measured. Consequently, the proposed EPS/pellicle mechanism, iron-mediated ecological filtering, calcium-associated matrix features, and potential process implications should be regarded as testable hypotheses rather than established causal or engineering properties. Validation will require functional genetics and polymer characterization, replicated matched cultivation experiments, strain-level biosafety assessment including virulence and antimicrobial-resistance determinants, and scale-up studies addressing mixing, oxygen transfer, biomass recovery, and process economics.

## 5 Conclusion

*Pseudomonas* sp. ABC1 combines a cohesive pellicle phenotype with extensive nutrient-acquisition capacity, a large cyclic-di-GMP regulatory repertoire, candidate non-canonical extracellular-polysaccharide loci, and persistence within a mixed open-culture consortium. The principal advance of this study is the integrated identification of biological features and candidate mechanisms that can now be tested through functional genetics, chemical characterization, community-resolved experiments, and controlled process studies.

These findings support ABC1 as a discovery-stage ecological model for investigating self-organization and biosynthetic persistence under open cultivation. They do not establish a validated fermentation process, equivalence or superiority relative to sterile cultivation, reduced sterilization or downstream-processing costs, superior productivity, contamination resistance, confirmed biomineralization, or industrial utility. Production kinetics were not measured, and the 7-day sampling point was selected for endpoint characterization; it should not be interpreted as an optimal fermentation duration.

Validation will require replicated and matched sterile-versus-open cultivations with time-resolved measurements of growth, substrate conversion, product identity and titre, yield, volumetric productivity, process variability and stability, community dynamics, biomass-recovery performance, biosafety, oxygen transfer, mixing, scale-up, and process economics.

## Data Availability

The complete genome sequence of *Pseudomonas* sp. ABC1 is available in NCBI RefSeq under accession GCF_013395055.1. Processed comparative-genomics, TonB, cyclic-di-GMP, EPS-locus, and functional-consortium tables accompany the revised Supplementary Material. The 16S rRNA gene amplicon sequence dataset is available in the NCBI BioProject repository with number PRJNA1514212. It can be accessed via the following URL: https://www.ncbi.nlm.nih.gov/sra/PRJNA1514212.
