# Supplementary material for: Biological process engineering by Pseudomonas sp. ABC1: a discovery-stage ecological chassis for open bioproduction
Source: Front Bioeng Biotechnol. 2026 Oct 26;14:1926795. doi: 10.3389/fbioe.2026.1926795 (PMC13745556; doi:10.3389/fbioe.2026.1926795)
Supplement: Supplementary file 1 [file Supplementaryfile1.docx]

Supplementary Material
Biological Process Engineering by Pseudomonas sp. ABC1: A Discovery-Stage Ecological Chassis for Open Bioproduction

**Contents.** This Supplementary Material contains Supplementary Tables S1-S2 and Supplementary Figures S1-S4. Supplementary Table S2 consolidates the processed comparative-genomics, TonB-dependent receptor, EPS-locus, Wzx/Wzy-candidate, and genome-informed consortium analyses into a single Excel workbook (ABC1_Supplementary_Table_S2_Combined.xlsx) organized into seven data sections.

# Supplementary Tables

**Table S1.** De novo peptide sequences identified by LC-MS/MS analysis of the open-culture peptidome. The table contains the complete peptide-level output used to summarize the 1,518 de novo sequences and 1,478 non-redundant peptide sequences reported in the manuscript.

**Table S2.** Processed comparative-genomics and genome-informed functional analyses of Pseudomonas sp. ABC1 and the open-culture consortium. The complete dataset is supplied as a single Excel workbook with seven sections (worksheets), as follows:

**Section A:** Curated TonB-dependent receptor repertoire of Pseudomonas sp. ABC1. Forty-six stringently annotated receptor proteins are reported, including the pyoverdine-receptor-like subset used in the main text.

**Section B:** Comparative conservation and maximum local colocalization of the reconstructed ABC1 EPS-associated loci PSL_140 and PSL_143 across the comparative genome set.

**Section C:** Protein-level best-hit amino-acid identity matrix for proteins encoded within PSL_140 and PSL_143 across comparator genomes.

**Section D:** Candidate Wzx/Wzy-associated membrane proteins within PSL_143, including predicted transmembrane topology, Pfam architecture, product annotation, candidate score, and supporting evidence.

**Section E:** Genome-inferred functional counts normalized per 1,000 predicted proteins for representative taxa from the open-culture consortium.

**Section F:** Abundance-weighted functional potential obtained by weighting normalized genome-inferred functional profiles by genus-level relative abundance derived from the 16S rRNA amplicon profile.

**Section G:** Functional complementarity indices for the reconstructed consortium. For each functional module, the complementarity index is defined as one minus the abundance-weighted fraction contributed by the dominant representative taxon.

**Interpretive note.** PSL_140 and PSL_143 are internal identifiers assigned to reconstructed ABC1 polysaccharide-associated loci and do not denote the canonical Pseudomonas psl operon. The consortium analyses represent genome-inferred functional potential and should not be interpreted as evidence of in situ transcription, metabolic flux, or direct metabolite exchange. The manuscript reports 29 cyclic-di-GMP-associated proteins in aggregate (24 GGDEF, 4 EAL, and 1 HD-GYP; Figure 3C); an individual per-protein cyclic-di-GMP table is not included in Supplementary Table S2.

# Supplementary Figures

**Figure S1.** Experimental workflow and downstream characterization. (A) Open, non-sterile cultivation of Pseudomonas sp. ABC1 in 5-L plastic bioreactor vessels, performed in triplicate under static conditions. (B) Pellicles were harvested manually after seven days of cultivation and transferred to Petri dishes for endpoint characterization. (C) Analytical techniques and multi-omics approaches used for pellicle characterization, including microscopy/EDX, 16S rRNA amplicon profiling, spectroscopic analysis, peptidomics/proteomics, and comparative genomics.

**Figure S2.** Micrographs and EDX elemental analysis of the pellicle and calcium-enriched structures. (A, B) SEM images showing the surface morphology of the bulk pellicle matrix. (C) EDX spectrum and elemental map illustrating calcium distribution across the pellicle surface. (D, E) SEM images of selected calcium-enriched structures embedded within the matrix. (F) EDX spectrum and elemental map of a representative localized structure showing elevated calcium (Ca). EDX data are interpreted as elemental evidence and do not establish mineral phase or biological causality.

**Figure S3.** Endpoint composition of the pellicle-associated microbial community during non-sterile open cultivation. Partial 16S rRNA gene sequencing after 7 days detected Pseudomonas, Delftia, Flavobacterium, Acidovorax, and additional environmental genera. The profile documents coexistence within a mixed pellicle community; temporal succession, causal ecological selection, and strain-level interactions were not resolved by this endpoint analysis.

**Figure S4.** Excitation and emission spectra of fluorescent compounds in the open-culture extracellular fraction. Steady-state photoluminescence profiles provide an optical fingerprint of the culture broth. Comparable spectral features in axenic and mixed-culture measurements indicate persistence of the characteristic fluorescent phenotype but do not establish metabolite identity, titre, or taxonomic origin.

**Abbreviations:** ABC, ATP-binding cassette; ASV, amplicon sequence variant; DGC, diguanylate cyclase; EAL, cyclic-di-GMP phosphodiesterase-associated domain; EDX, energy-dispersive X-ray spectroscopy; EPS, extracellular polysaccharide; GGDEF, diguanylate cyclase-associated domain; GT, glycosyltransferase; HD-GYP, cyclic-di-GMP phosphodiesterase-associated domain; LC-MS/MS, liquid chromatography-tandem mass spectrometry; PSL_140 and PSL_143, internal identifiers for reconstructed ABC1 polysaccharide-associated loci; SEM, scanning electron microscopy; TM, transmembrane; Wzx, putative polysaccharide repeat-unit flippase/translocase; Wzy, polysaccharide repeat-unit polymerase.
